# Diagnostic and therapeutic significance of selected antibodies against advanced glycation end-products (anti-AGEs)

**DOI:** 10.1007/s11033-026-12545-9

**Published:** 2026-08-04

**Authors:** Julia Sobczyńska, Jakub Federowicz, Zuzanna Galińska, Maciej Ziomek, Wojciech Stach, Agnieszka Bronowicka-Szydełko, Katarzyna Madziarska

**Affiliations:** 1 Faculty of Medicine, Wroclaw Medical University, Wroclaw, Poland; 2 Wroclaw Medical University, Wroclaw, Poland; 3 Department of Biochemistry and Immunochemistry, Wroclaw Medical University, Wroclaw, Poland; 4 Clinical Department of Diabetology, Hypertension and Internal Diseases, Institute of Internal Diseases, Wroclaw Medical University, Wroclaw, Poland

**Keywords:** diabetes, glycation, Advanced Glycation-End products (AGEs), antibodies

## Abstract

The aim of this review is to summarize current knowledge on the diagnostic and therapeutic significance of antibodies against advanced glycation end-products (anti-AGEs). AGEs are formed through non-enzymatic reactions of reducing sugars with proteins, lipids, and nucleic acids, and their accumulation has been implicated in the pathogenesis of chronic diseases such as diabetes, atherosclerosis, cancer, and neurodegenerative disorders. This paper discusses anti-MGO modified proteins, anti-imidazolone, anti-pentosidine, anti-CML (including SIWA318H), anti-CEL, anti-CMA, and anti-GA-pyridine antibodies, with a focus on their potential applications in diagnostics (biomarkers, disease progression monitoring) and therapy (e.g., SIWA318H in pancreatic cancer). The review also highlights current limitations, particularly regarding clinical use in humans, and outlines perspectives for further research in this promising field.

## Introduction

Advanced glycation end-products (AGEs) are a heterogeneous group of substances whose significance has been increasing lately due to growing evidence for their role in pathogenesis of multiple diseases [1]. They are products of spontaneous, non-enzymatic reactions between reducing sugars (e.g., glucose, fructose) and various proteins, lipids or nucleic acids throughout the body. The higher the serum levels of saccharides, the more effectively the glycation process occurs (e.g. in hyperglycemic states and diabetes). Factors such as aging, oxidative stress, and inflammation also induce AGEs formation. Serum levels of AGEs are currently mostly used for research purposes, with potential in monitoring and prognosing course of certain diseases [2]. Examples of AGEs include, 3-DG-imidazolone, Nε-(Carboxymethyl)lizyne (CML), Nε‑(Carboxyethyl)lysine (CEL), N(omega)-carboxymethyl-arginine (CMA), and GA-pyridine [3]. Although methylglyoxal (MGO) is not classified as an AGE, it is a highly reactive dicarbonyl compound that plays a central role in non-enzymatic glycation and AGE formation. For this reason, MGO-derived modifications are also included in this review.

An important distinction needs to be made between glycation and glycoxidation process. Glycation refers to the non-enzymatic reaction of reducing sugars with amino groups, which initially proceeds without oxygen. On the other hand, glycoxidation describes the oxidative reactions that occur during later stages of glycation, leading to irreversible AGEs such as CML or pentosidine. Thus, glycoxidation is a component of glycation, not an independent process [4, 5]. 

It is important to emphasize that not only saccharides can be involved in non-enzymatic spontaneous reactions. Other substrates, such as lipids, may also undergo a similar process resulting in lipoxidation end-products (ALE). Those agents may activate similar pathways to those of AGEs, but their synthesis process and structure is quite different [4, 6]. 

**Fig. 1:**
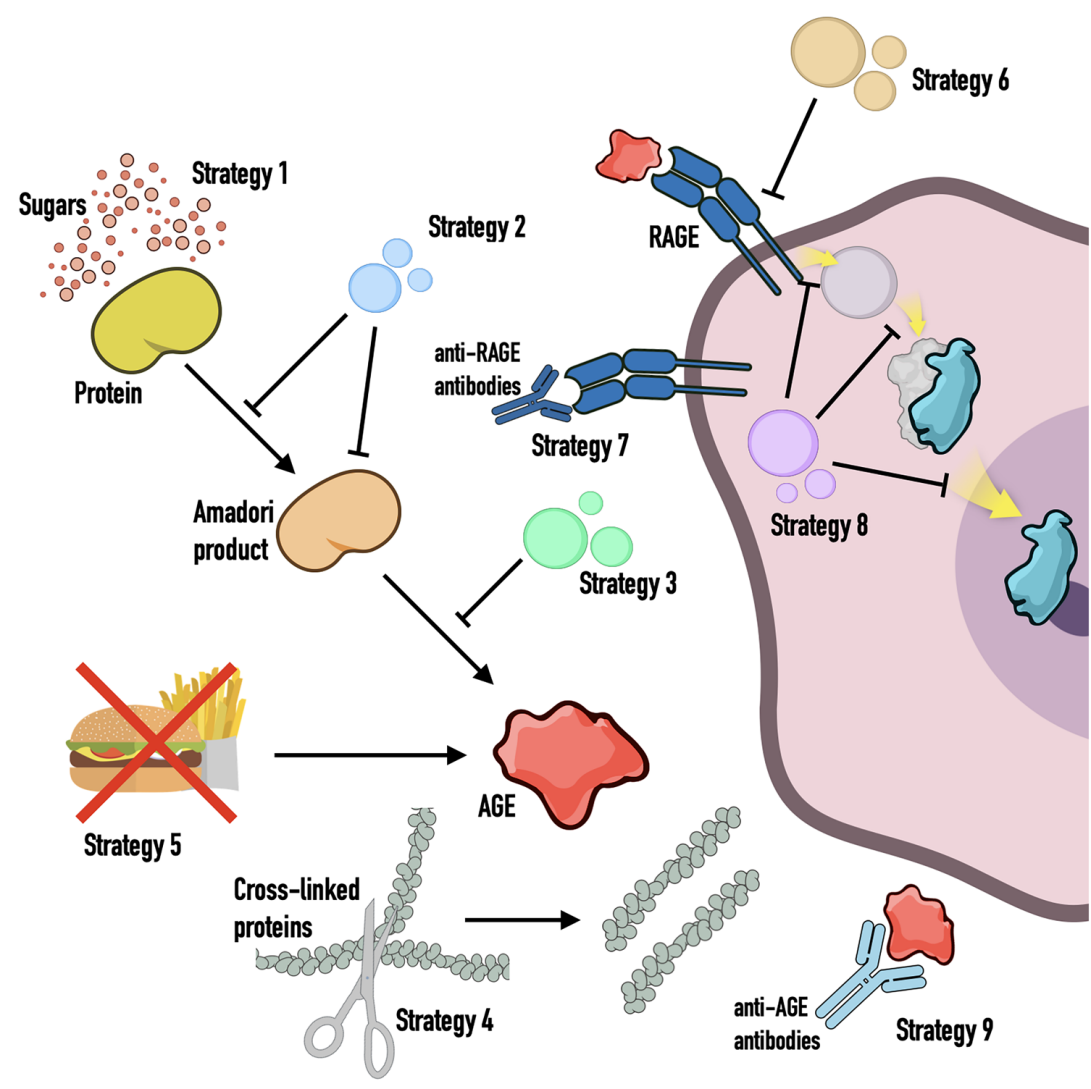
Possible AGE clearance strategies: (1) reduction of AGE accumulation by strict glycemic control; (2) interference with the formation or persistence of Amadori products; (3) suppression of AGE formation; (4) destruction of pathological cross-links between AGE and structural proteins; (5) lowering dietary intake of AGE rich food; (6) interference with AGE receptor signaling; (7) immunological blockade of RAGE activation; (8) suppression of intracellular pathways; (9) AGE neutralization using anti-AGE antibodies. Abbreviations: AGE: advanced glycation end-product; RAGE: receptor for advanced glycation end-products

A number of endogenous mechanisms have been identified that limit the formation and accumulation of advanced glycation end products (AGEs). These include low-molecular-weight antioxidants such as pyruvic acid, bilirubin, and glutathione, as well as enzymatic defense systems comprising superoxide dismutase, catalase, glutathione peroxidase, glutathione reductase, heme oxygenase, and thioredoxin [7–9]. Beyond intrinsic protective systems, several therapeutic approaches have been proposed to counteract AGEs generation or attenuate their biological consequences. The proposed mechanisms are illustrated in Fig. 1.

### Strategy 1. Regulation of glucose homeostasis

Maintaining strict glycemic control reduces substrate availability for nonenzymatic glycation reactions and consequently limits AGEs deposition, particularly within vascular structures. This approach, however, does not prevent AGEs formation mediated by reactive carbonyl species or other reducing sugars independent of glucose [10]. 

### Strategy 2. Modulation of early Maillard reaction intermediates

**Fig. 2:**
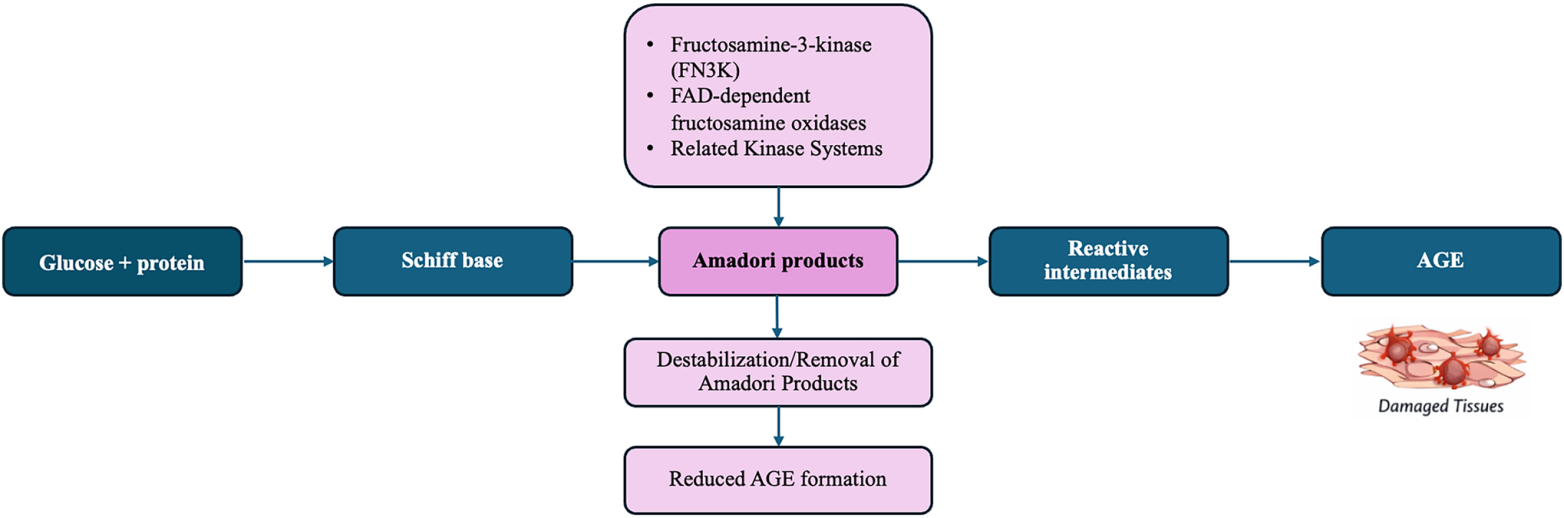
Strategy 2: Modulation of early Maillard reaction intermediates

Interfering with the formation or persistence of Amadori products represents another preventive approach. Endogenous deglycation pathways have been described in bacteria, fungi, and mammals. These involve FAD-dependent fructosamine oxidases, ATP-dependent fructosamine-3-kinases (FN3K), and related kinase systems that modify or destabilize glycated intermediates. Enhancing these enzymatic pathways may reduce progression toward irreversible AGEs formation [11]. **See** Fig. 2.

### Strategy 3. Pharmacological suppression of AGEs synthesis

Various carbonyl-scavenging agents and Maillard reaction inhibitors, including aminoguanidine, pyridoxamine, LR-90, OPB-9195, and ALT-946, have been investigated. These compounds primarily act by neutralizing reactive carbonyl intermediates or interrupting late glycation steps [12, 13]. While experimental data suggest protective effects in diabetic complications, clinical translation has been limited by safety concerns [14]. 

### Strategy 4. Disruption of AGE-induced protein cross-linking

Certain thiazolium derivatives, such as alagebrium (ALT-711) and N-phenacylthiazolium bromide, are capable of cleaving pathological cross-links formed between AGEs and structural proteins. By reducing protein cross-linking, these agents may improve tissue elasticity and vascular compliance without disturbing physiological glycosidic bonds [15, 16]. Although initial human trials in patients with heart failure were promising, showing reduced left ventricle mass and improved diastolic function [15, 17]. Subsequent BENEFICIAL placebo-controlled clinical trial demonstrated that alagebrium (ALT-711) did not improve exercise tolerance or cardiac function in patients with heart failure [18]. N-phenacylthiazolium bromide has also been studied in the literature in animal models, including rat studies describing its potential efficacy in treating vascular complications of diabetes or periodontitis [19, 20]. Additionally in ex vivo studies on human cancellous bone, N-phenacylthiazolium bromide significantly reduced AGEs content compared with controls and was associated with a significant improvement in post-yield material properties [21]. However, there is a lack of adequate in vivo studies in humans.

### Strategy 5. Minimization of exogenous AGEs burden

Lowering dietary intake of AGEs-rich foods, avoiding tobacco exposure, and maintaining regular physical activity have been associated with decreased systemic AGEs levels and reduced progression of glycation-related vascular stiffening [22]. 

### Strategy 6. Interference with AGEs’ receptor signaling

**Fig. 3:**
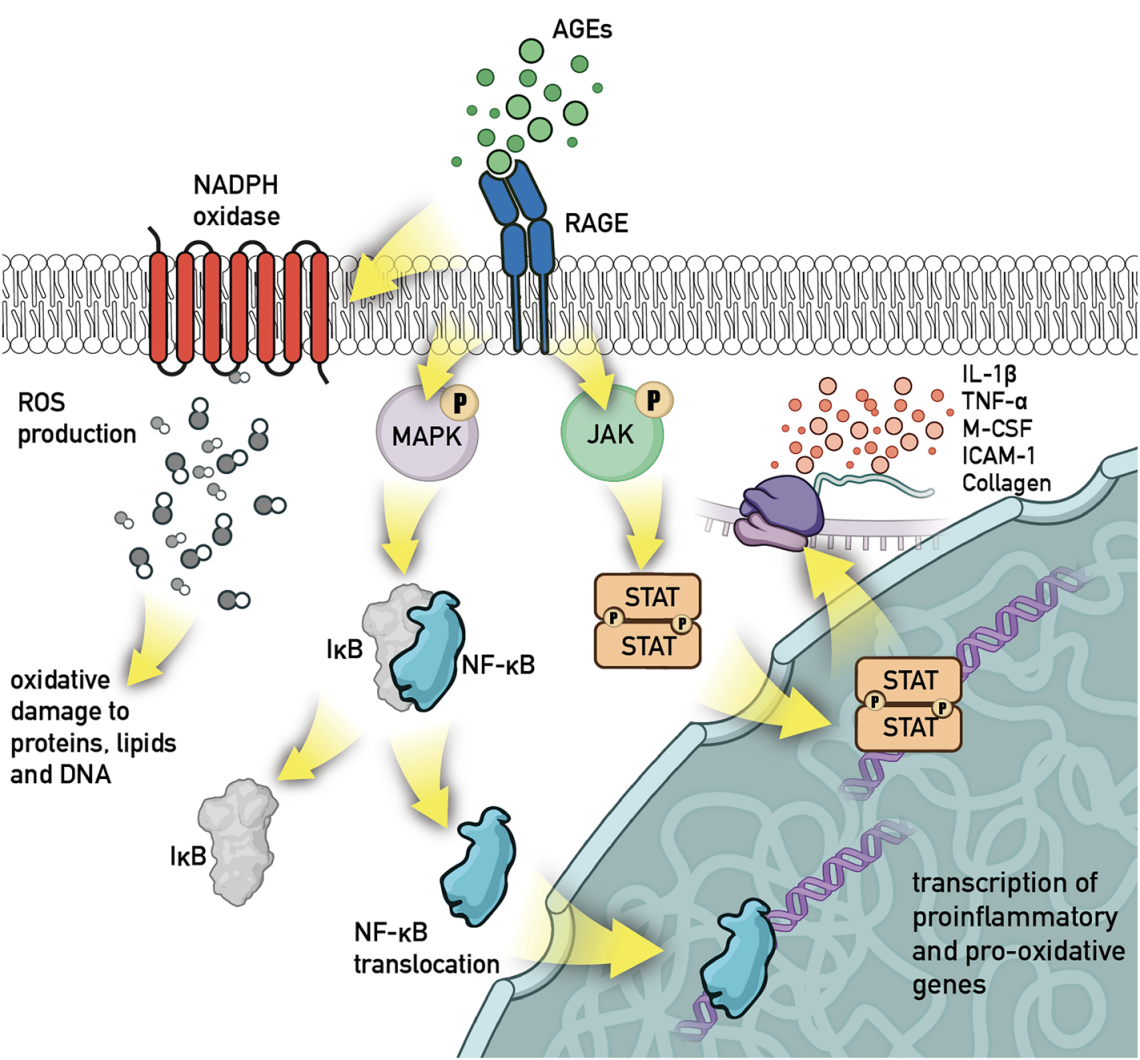
The mechanism of action of AGE-RAGE binding. The binding of AGEs with RAGE activates intracellular cascades. Activation of NADPH oxidase leads to an increase in ROS production, which cause oxidative damage to proteins, lipids and DNA. After binding to the ligand, RAGE phosphorylates its downstream MAPK and activates NF-κB protein. Phosphorylation of JAK kinase leads to phosphorylation of STAT, which then dimerizes. Both activated, NF-κB and STAT, enter the nucleus to promote the transcription of proinflammatory and pro-oxidative genes. Abbreviations: AGEs: advanced glycation end-products; RAGE: receptor for advanced glycation end-products; ROS: reactive oxygen species; MAPK: mitogen-activated protein kinase; NF-κB: nuclear factor kappa-B; IκB: inhibitor of NF-κB; JAK: Janus kinase; STAT: signal transducer and activator of transcription; IL-1: interleukin-1β; TNF-α: tumor necrosis factor α; M-CSF: macrophage colony-stimulating factor; ICAM-1: intracellular adhesion molecule 1

AGEs exert biological effects both independently of receptors and through receptor-mediated mechanisms [23]. The receptor for advanced glycation end products (RAGE) is the best-characterized mediator of AGEs-induced signaling [24]. Ligand binding to membrane-bound RAGE activates intracellular cascades, including MAPK pathways and protein kinase C, culminating in NF-κB nuclear translocation and transcription of proinflammatory and pro-oxidative genes. This mechanism is illustrated in Fig. 3. These processes enhance cytokine production, activating mast cells adhesion molecule expression, extracellular matrix remodeling, oxidative stress, and thrombogenic activity [25–30]. 

Several additional AGEs-binding proteins contribute to receptor-mediated responses, including AGE-R1, AGE-R2, AGE-R3 (galectin-3), and members of the ERM protein family (ezrin, radixin, moesin) [31]. Two distinct pools of soluble RAGE (sRAGE) contribute to decoy receptor function in the circulation. Endogenous secretory RAGE (esRAGE), generated through alternative splicing of AGER gene, is directly secreted as a soluble protein lacking the transmembrane and cytoplasmic domains. Additionally, proteolytic cleavage of membrane-bound full-length RAGE releases a soluble ectodomain, called cleaved RAGE (cRAGE). Total circulating sRAGE represents the combined pool of esRAGE and cRAGE. Both isoforms are capable of sequestering circulating ligands, thereby limiting their interaction with membrane-bound RAGE. However, their biological significance may differ - esRAGE is thought to represent a regulated protective mechanism, while elevated cRAGE may partially reflect increased RAGE expression and pathological shedding [32–35]. 

### Strategy 7. Immunological blockade of RAGE activation

An alternative approach focuses on preventing receptor activation through neutralizing antibodies directed against RAGE. Experimental evidence from animal models indicates that anti-RAGE antibody therapy reduces inflammatory signaling, decreases vascular permeability, limits atherogenesis, and slows the progression of diabetic nephropathy [36–39]. In addition, partial attenuation of fibrotic remodeling has been observed [40]. This strategy does not reduce AGEs formation per se but interrupts downstream pathological signaling initiated at the receptor level.

### Strategy 8. Suppression of downstream signal transduction pathways

Certain pharmacological agents, including selected bisphosphonates, statins, and curcumin, have been shown to inhibit intracellular pathways activated by AGE–RAGE interaction, thereby diminishing inflammatory and oxidative responses [41–43]. 

Clearance of AGE-modified macromolecules often occurs through receptor-mediated endocytosis and degradation within the endosomal–lysosomal system. Scavenger receptors facilitate internalization of AGE-containing complexes, followed by acidification-dependent dissociation and enzymatic degradation in lysosomes [44, 45]. Proteasomal degradation and autophagic mechanisms further contribute to the removal of glycated proteins.

### Strategy 9: Anti-AGEs antibody-mediated neutralization of advanced glycation end products

A ninth strategy may be distinguished. Although conceptually related to strategy seven, it differs in its mechanism of action. Strategy seven is based on antibody-mediated blockade of the RAGE receptor, whereas an alternative immunological approach involves direct binding of pre-formed AGEs, thereby preventing their interaction with RAGE and other cellular receptors. Consequently, the activation of pro-inflammatory signaling pathways is attenuated. In this study, we focus on the clinical implications of these antibodies.

Anti-AGEs are antibodies directed against AGEs, currently being investigated for their potential clinical applications. They include both autoantibodies, produced endogenously against glycation products formed within the body, and alloantibodies, obtained through immunization in animal models. In the further part of this review we will describe the specific potential of each antibody. Some of them may be used in diagnostics to detect tissue deposits or to measure serum levels of AGEs. Some may serve as a prognostic factor in cardiovascular diseases. For other anti-AGEs, increased levels in certain disorders suggest their involvement in disease pathogenesis. Last, but not least, Anti-AGEs may also have therapeutic potential in diseases such as pancreatic cancer, one of the most lethal malignancies with median overall survival between 8 and 11 months in metastatic cases [46]. We will focus on anti-methylglyoxal, anti-imidazolone, anti-pentosidine, anti-CML (including SIWA318H), anti-CEL, anti-CMA, and anti-GA-pyridine antibodies.

Anti-AGEs antibodies represent a heterogeneous group of molecules currently under investigation for potential clinical applications. Some of these antibodies directly target AGEs themselves, while others are directed against AGE-modified molecules, such as proteins. Their role is being discussed in the context of various chronic diseases **[see** Tables 1 and 2**].** Based on their origin and application, anti-AGEs antibodies can be divided into different categories - naturally occurring autoantibodies against AGEs, experimentally generated antibodies used to detect AGEs and experimentally generated therapeutic antibodies directed against AGEs.

**Table 1:** Examples of potential applications of anti-AGEs antibodies in various diseases

Disease	Potential role of Anti-AGEs antibodies	References
T2D	May be useful in patients with hypertension and type 2 diabetes (T2D) for assessing vascular risk. Anti-DNA-AGE antibodies may serve as an immunological marker in predicting the initiation and progression of vascular complications in these patients.	[47, 48]
Rheumatoid arthritis	Anti-AGEs antibodies are associated with radiological progression of RA and are present in seronegative patients with HLA-DRB1*03	[49]
Autoimmune hepatitis	Anti-PTM antibodies, including anti-AGEs, are present in patients with autoimmune liver disease (AILD) and may be used to predict treatment efficacy in AIH	[50]
Pancreatic cancer	The anti-AGE antibody SIWA318H has proven effective in preclinical studies on pancreatic cancer treatment	[51]
CAD, COPD	Anti-AGEs antibodies may be used to determine COPD risk in patients with coronary artery disease (CAD) undergoing CABG	[52]
Alzheimer Disease	Level of IgM autoantibodies against a CEL-modified apolipoprotein A1 (ApoA1^141–147) peptide level may be used as early indicator of a disease	[53]

## Naturally occurring autoantibodies against AGEs

### Anti-methylglyoxal

Methylglyoxal (MGO) is a highly reactive α-oxoaldehyde generated endogenously through both enzymatic and non-enzymatic pathways. It modifies lysine residues—resulting in the formation of Nε-(carboxyethyl)lysine (CEL), the immunological significance of which is discussed in a separate section—as well as arginine residues within proteins, leading to the formation of various advanced glycation end-products (AGEs) [54]. Histones are also susceptible to MGO-induced modifications [55, 56]. These MGO-derived AGEs have been implicated in the pathogenesis of several chronic diseases, including diabetes and cardiovascular disorders. This review includes a discussion of the role of antibodies targeting MGO-modified proteins.

Protein glycation, including glycoxidation reactions, is highlighted in the development of biomarkers for early detection of cancer. Carcinogenesis is associated with increased oxidative stress, which induces various molecular changes, including the glycoxidation of histone H2A by methylglyoxal. The specificity of antibodies against modified histones was investigated in 96 patients aged 15–63 years with different types of cancers, including cancers of the head and neck region, ovary, breast, and colon, and compared with a control group consisting of 35 healthy individuals. Patient sera were analyzed for the presence of autoantibodies against both native H2A and glycoxidation-modified MG-H2A histones. The study demonstrated that epitopes generated on the MG-modified H2A are significantly more immunogenic than those on the native molecules. This leads to the production of highly specific circulating IgG antibodies, which may potentially be utilized in the future for the development of biomarkers for early cancer detection [55]. However, the diagnostic accuracy of these antibodies for discriminating cancer patients from healthy controls - quantified by sensitivity, specificity or ROC analysis - was not reported. Without such analysis, and without validation in a cohort including patients with benign inflammatory conditions that may also increase oxidative stress and AGEs formation, the clinical utility of these antibodies as a screening tool remains hypothetical. Furthermore, this conclusion is drawn from a single study with a relatively small cohort (*n* = 96) and a control group (*n* = 36) that may not fully account for confounding factors such as glycemic status or renal function, both of which affect AGEs levels. Further validation in larger, multi-center cohorts is required to confirm diagnostic specificity.

A protective role of anti-MGO-B100 IgM antibodies in reducing the risk of coronary disease progression in patients with type 2 diabetes is also hypothesized. A cohort study involving 497 patients with type 2 diabetes demonstrated that higher titers of these antibodies were associated with less severe coronary disease. The severity of coronary disease was assessed using coronary artery calcium (CAC) imaging. Interestingly, patients with a family history of premature myocardial infarction exhibited lower levels of anti-MGO-B100 IgM, suggesting a potential influence of genetic factors in the regulation of these antibodies. Based on these findings, a protective role of anti-MGO-B100 IgM in diabetic vasculopathy is postulated. No such associations were observed in relation to anti-MGO-B100 IgG [57]. In a subsequent study, it was demonstrated that the immune-dominant epitopes of MGO-modified apoB100 include peptide 220 (p220), and antibodies targeting this epitope are specific. The levels of these antibodies, in both IgG and IgM classes, were then measured in 700 middle-aged participants from the prospective cohort of the Malmö Diet and Cancer (MDC) study. Participants who experienced a cardiovascular event during the follow-up period exhibited lower levels of anti-MGO-p220 IgM antibodies [58]. Furthermore, low levels of these antibodies were associated with an increased risk of cardiovascular events even among individuals without diabetes. As in the previous study, no such association was observed for IgG-class antibodies [57]. A key strength of these studies is the use of a prospective cohort allowing for the assessment of predictive value. However, the exclusive protective association observed with the IgM isotype, but not IgG, remains mechanistically unexplained and highlights a critical gap in our understanding.

### Anti-CEL

Nε‑(Carboxyethyl)lysine (CEL) is an advanced glycation end-product formed by the non-enzymatic reaction between lysine and methylglyoxal. Anti‑CEL autoantibodies are detectable in human plasma at high titers [59, 60]. They exhibit strong specificity for CEL-modified proteins with no cross-reactivity to structurally similar AGEs adducts such as CML, CMA, or CMC [59]. Functionally, autoanti‑CEL antibodies enhance macrophage uptake of CEL-modified proteins in vitro and promote their hepatic accumulation in vivo, suggesting a role in AGEs clearance [60]. 

Autoantibodies targeting Nε‑(carboxyethyl)lysine (anti‑CEL) have been observed in a range of chronic conditions, highlighting their potential role in disease pathogenesis and immune regulation. In patients with hidradenitis suppurativa (HS), anti‑CEL antibodies of the IgG, IgA, and IgM classes are significantly elevated, with IgG2 being the dominant subclass. CEL-specific plasmablasts have been isolated directly from skin lesions, and serum antibody levels correlate with both disease severity and duration. These findings suggest that anti‑CEL responses may contribute to local inflammation and tissue damage in HS [61]. 

In Alzheimer’s disease, a Taiwanese study on 32 patients reported increased levels of IgM autoantibodies against a CEL-modified apolipoprotein A1 (ApoA1^141–147) peptide. The highest titers were observed in the early stages of the disease and were associated with better cognitive scores, raising the possibility that these antibodies could serve as early biomarkers or reflect a protective immune response [53]. However, the cross-sectional comparison of established diagnostic groups does not allow assessment of predictive performance in a pre-symptomatic population. A clinically useful Alzheimer’s biomarker must be evaluated in a longitudinal, prospective cohort to determine whether elevated antibody levels precede cognitive decline and to calculate sensitivity and specificity at various cut-off points.

## Experimentally generated antibodies used to detect AGEs

### Anti-imidazolone

3-DG-imidazolone compounds are a product of a reaction between arginine derivative with 3-deoxyglucosone (3-DG). At first 3-DG attacks arginine residues in protein and then reacts with lysine under physiological conditions [62]. Among other AGEs created by incubation with 3-DG such as CML, pyrraline and pentosidine, imidazolone is the most specific marker of 3-DG protein modification of tissue protein in vivo [63]. Monoclonal antibodies against imidazolone have been created by immunizing mice with AGE-modified keyhole limpet hemocyanin (AGE-KLH), which lead to creation of two monoclonal clone types of anti-AGEs antibodies (AG-1 and AG-10). The epitope of AG-1 has been found to be imidazolone [62]. 

As the formation of 3-DG occurs under non-oxidative conditions, presence of imidazolone modified proteins may reflect on the non-oxidative pathway of AGEs formation. This contrasts with glycoxidation products such as CML and pentosidine, which require oxidative steps. This allows researchers to differentiate non‑oxidative from oxidative AGEs generation pathways, aiding pathophysiologic profiling [64]. 

ELISA with AG-1 can be used to measure erythrocyte levels of imidazolone. Research has shown that erythrocytes levels of imidazolone are significantly higher in patients with diabetes compared to nondiabetic patients [65]. 

Using the AG-1, imidazolone has been detected in the glomeruli from the diabetic patients, whereas immunostaining of nondiabetic patients showed no significant deposit of imidazolone in the glomeruli. Showing that nodular lesions of imidazolone in the glomeruli are characteristic for patients with diabetic nephropathy. Imidazolone has also been found in atherosclerotic aortic tissues from both diabetic and nondiabetic patients, which proves AGEs, such as imidazolone, are associated with atherosclerotic lesions regardless of the presence of diabetes. However, atherosclerotic lesions with presence of imidazolone have been found in younger patients suffering from diabetes and uremia than in those without diabetes and uremia. This suggests that those diseases play a part in accelerating the development of atherosclerosis in patients [62]. 

The reviewed studies demonstrate that anti-imidazolone antibodies are effective for immunohistochemical detection in tissues, and that imidazolone may be a contributing factor to progression of long-term diabetic complications such as nephropathy and atherosclerosis. However, the lack of quantitative clinical cohort studies limits their application. Without this level of clinical validation, the antibody remains a powerful research reagent, but cannot be considered a clinically validated biomarker.

### Anti-pentosidine

Pentosidine is formed in the reaction of the amino acids and Maillard reaction products of pentoses. Although pentosidine can be synthesised using many different pentoses, ribose seems to be the most reactive one [66]. These AGEs form fluorescent cross-links between the arginine and lysine residues in collagen. Pentosidine is present in serum both in free and albumin-linked form [67]. 

Anti-pentosidine antibody has been created by immunizing New Zealand White rabbits with keyhole limpet hemocyanin conjugate with pentosidine (KLH-pentosidine). Antibodies formed this way strongly react with pentosidine, but not with pentosidine-like compounds [68]. 

Researchers have used anti-pentosidine antibodies to detect pentosidine deposits in different tissues and in various diseases such as diabetic nephropathy [69], Alzheimer’s disease [70], pulmonary hypertension [71] or age-related inclusions in the human brain [72]. Immunohistochemical staining shows promise as a useful tool in research of causes, pathology and complications of many diseases.

Using anti-pentosidine antibodies, ELISA systems have been created, which allow to reliably measure plasma and urinary levels of pentosidine [73, 74]. Using those systems serum pentosidine levels have been measured and correlated with arterial stiffness in hemodialysis patients [75] and urinary levels are significantly associated with grip strength and gait speed [76]. While the correlation of serum and urinary pentosidine levels with functional outcomes is promising, these studies are largely cross-sectional. This design limits the ability to infer causation or determine the relationship between pentosidine accumulation and functional decline. The clinical value of measuring pentosidine as a biomarker would be substantially strengthened by longitudinal studies that establish the predictive value of these measurements and their utility for monitoring disease progression or therapeutic response.

### CML

Nε-(Carboxymethyl)lysine (CML) is a modified amino acid formed through a non-enzymatic reaction. It belongs to a group of advanced glycation end-products (AGEs), which are ligands for the receptor RAGE. CML is one of the final products of advanced glycation. It is typically found within proteins such as albumin or collagen and can be located both intracellularly and extracellularly. Elevated levels of CML have been associated with diabetes [77] and aging [78]. In addition, CML-modified collagen has been shown to induce apoptosis in fibroblasts [79]. CML is the major antigenic AGEs compound [80]. 

Monoclonal antibodies targeting Nε-(carboxymethyl)lysine (CML) and CEL represent essential tools for the investigation of advanced glycation end products (AGEs), facilitating the detection and characterization of CML-modified and CEL-modified proteins in diverse biological and pathological contexts [81]. CML-protein and CEL-protein adducts constitute an important immunological epitope.

The antibody clone 6D12 doesn’t exhibit high specificity and strong immunoreactivity toward a range of CML-conjugated proteins, it also detects CEL-conjugated proteins. Notably, the presence of a carbonyl group is critical for 6D12 binding [82, 83]. This progressive refinement of antibody specificity underscores a critical caveat in interpreting the historical literature on CML. Findings from studies that did not explicitly control for cross-reactivity may reflect the combined detection of CML and structurally similar epitopes like CEL.

To overcome cross-reactivity limitations observed in earlier antibodies such as 6D12, the monoclonal antibody CMS-10 was developed to selectively recognize CML without cross-reacting with structurally related epitopes such as Nε-(carboxyethyl)lysine (CEL). In this context, the monoclonal antibody 2D6G2 demonstrated robust and selective binding to CML-modified BSA and KLH, with no detectable reactivity toward native, unmodified proteins. A competitive ELISA employing 2D6G2 was established for the quantification of serum CML levels in patients with chronic kidney disease (CKD), revealing a clear correlation between CML concentrations and CKD stage. These findings underscore the potential utility of 2D6G2 as a sensitive and specific reagent for clinical assessment of AGE-associated toxicity [80]. 

### Anti-CMA

N(omega)-carboxymethyl-arginine (CMA) is a structurally defined advanced glycation end-product (AGEs) classified under AGE5, a subgroup of AGEs derived from glyoxal (GO-AGEs). Among the five major AGEs’ categories CMA represents a key modification resulting from the reaction between glyoxal and the guanidino group of arginine residues in proteins. Studies have demonstrated that CMA levels are significantly elevated in individuals with diabetes compared to non-diabetic controls, indicating its relevance in hyperglycemic states and its possible contribution to diabetes-related complications. CMA formation has been particularly associated with the non-enzymatic glycation of long-lived proteins such as collagen [84]. Due to its specific formation on arginine residues within collagen, monoclonal antibodies against CMA could serve as diagnostic tools for identifying glycation-related tissue remodeling.

A monoclonal antibody specific for CMA was prepared to detect histological localization of CMA. The antibody was confirmed to be specifically reactive only to the CMA structure by ELISA, indicating its potential utility for the detection of CMA in glycated proteins. The CMA-recognition capacity of the monoclonal anti-CMA antibody was examined using immunohistochemistry on atherosclerotic tissue obtained from non-diabetic patients. Vascular specimens, including sections from the aortic wall and internal carotid artery, were analyzed from three individuals, all exhibiting fibroatheromatous changes, some of which showed evidence of ulcerative features. The results showed that CMA immunoreactivity was prominently detected in atherosclerotic lesions, while no such reactivity was observed in the surrounding healthy tissue. Additionally, the staining intensity of the anti-CMA antibody was significantly reduced when pre-incubated with CMA-conjugated HSA, indicating specificity of the antibody-antigen interaction. These findings support the hypothesis that the accumulation of CMA-modified proteins may play a role in the pathophysiology of aging and related diseases, including atherosclerosis [85]. However, the results should be interpreted with caution. The study was based on tissue samples obtained from only three patients, and antibody specificity was evaluated primarily by ELISA and inhibition assays. Further validation using complementary analytical methods and studies involving larger patient cohorts would be valuable to confirm the histological detection of CMA and clarify its potential association with atherosclerosis.

### Anti-GA-pyridine

GA-pyridine is a cytotoxic advanced glycation end product (AGEs) formed from the reaction of glycolaldehyde (GA) with proteins. Among GA-derived AGEs, GA-pyridine constitutes a structurally distinct and antigenic adduct, reflecting GA-specific protein glycation. Immunohistochemical analyses have demonstrated the accumulation of GA-pyridine within cytoplasm of foam cells and in the central region of atheroma in atherosclerotic lesions, a phenomenon that may be associated with the pathogenesis of osteoarthritis (OA) [86–89]. Given that AGEs disrupt extracellular matrix integrity by modifying protein structure and function, it is hypothesized that GA-pyridine contributes to the progression of OA. In this study, not only GA-pyridine but also other AGEs structures potentially implicated in OA were examined, using epitope-specific antibodies that enabled precise localization of individual AGEs species within degenerated cartilage. These antibodies may therefore serve as valuable tools for elucidating AGEs-related mechanisms in degenerative joint disease and for advancing our understanding of the molecular pathways driving OA progression [87, 88]. 

Specific antibodies were generated, including a monoclonal antibody against GA-pyridine (clone 2A2). Osteochondral specimens from the medial tibial plateau were obtained during routine total knee arthroplasty from nine patients with clinically and radiographically confirmed knee osteoarthritis (OA). Histopathological assessment of cartilage degeneration was performed using the Osteoarthritis Research Society International (OARSI) cartilage histopathology grading system. Immunoreactivity (IR) was evaluated semi-quantitatively by calculating the staining H-score, which integrates both the extent and intensity of staining within the target tissue.

OA progression was characterized by a concomitant increase in GA-pyridine and RAGE immunoreactivity, demonstrating a strong association between these two markers. These findings suggest that GA-pyridine may represent one of the most reactive AGEs species capable of interacting with RAGE, thereby contributing to cartilage degeneration [87]. However, these findings should be interpreted with caution, as the study included a relatively small number of patients, all of whom had advanced OA requiring total knee arthroplasty, which may limit the generalizability of the results. The antibody appears to be a useful tool for investigating the distribution of GA-pyridine in osteoarthritic cartilage and exploring its potential role in OA pathogenesis. However, additional studies involving larger patient cohorts are needed to confirm these observations.

At present, clinical validation of the anti-GA-pyridine antibody remains limited, particularly regarding its diagnostic sensitivity and specificity, which restricts its application mainly to research settings rather than clinical use.

### Distinguishing free AGEs adducts from protein-bound and crosslinked AGEs

A critical nuance that affects the interpretation of nearly all studies using anti-AGEs antibodies is whether the detected antigen represents a free AGEs adducts, a non-crosslinking modification on a protein or a crosslinking structure between proteins. This distinction has profound consequences for understanding pathophysiology. Free AGEs are metabolic waste products largely reflecting renal clearance and protein turnover, while protein-crosslinked AGEs are cumulative, irreversible markers of long-term tissue damage and drivers of structural dysfunction [90, 91]. Crucially most common monoclonal antibodies, including anti-imidazolone, anti-pentosidine and anti-CML, bind the modified epitope irrespective of its molecular context. Therefore, these antibodies do not differentiate between free adducts, protein-bound and crosslinked AGEs [62, 68, 80]. 

The discrimination between these forms is achieved exclusively through the experimental design and assay format. Immunohistochemistry on fixed tissue sections primarily visualizes protein-bound and crosslinked AGEs. This is because free AGEs adducts are washed away during the tissue fixation and staining process. These findings are therefore indicative of local, accumulated tissue damage [92]. Whereas, ELISA systems can be configurated to measure different compartments. A direct or competitive ELISA on untreated serum or urine, as used to quantify pentosidine, measures the total pool of a specific AGE epitope, encompassing both free and peptide-bound fractions [73, 74]. Conversely, a sandwich ELISA using a capture antibody against specific protein backbone and an anti-AGE detection antibody will exclusively measure AGEs on that specific protein e.g. AGEs-modified Apolipoprotein A-I [93]. 

For future research, it is imperative that studies explicitly report not only the antibody clone and it’s target epitome, but also the molecular for of the antigen being measured - free AGEs adducts, total protein-bound AGEs, AGEs on a specific named protein or tissue-immobilized AGEs. This precision is essential to harmonize findings across studies.

## Experimentally generated therapeutic antibodies directed against AGEs

### SIWA318H

In our study, we primarily focus on the numerous diagnostic applications of anti-AGEs antibodies. However, an important question arises: is there also potential for therapeutic use in this field? A recent study on the anti-AGE antibody SIWA318H in the treatment of pancreatic cancer suggests that this may indeed be the case. As is widely recognized, pancreatic cancer remains one of the most challenging malignancies, both in terms of diagnosis and treatment [94, 95]. 

SIWA318H is an anti-CML antibody, but we have chosen to discuss it in a separate section due to its potential therapeutic relevance. From a biochemical standpoint, SIWA318H is a humanized IgG1-class antibody.

In a preclinical mouse model (immunodeficient NSG mice engrafted with human CD34 + hematopoietic stem cells), SIWA318H was shown to bind to pancreatic cancer cells, cancer-associated fibroblasts, as well as to patient-derived xenografts. In the latter case, the antibody significantly slowed tumor progression in treated mice compared to the control group. Moreover, a significant increase in complete remission (CR) was observed in the treatment group (60% in the high-dose group and 77.8% in the low-dose group, vs. 6.7% in the control group). It was also demonstrated that SIWA318H induces antibody-dependent cell-mediated cytotoxicity (ADCC) targeting pancreatic cancer cells. The results of this study, including a 60-77.8% complete remission rate, must be interpreted with caution. The control group received no treatment, which does not reflect the clinical reality where patients would receive standard-of-care chemotherapy (e.g. FOLFIRINOX or gemcitabine/nab-paclitaxel). Therefore, it remains unknown whether SIWA318H is superior to, equivalent to, or synergistic with existing therapies.

One of the major obstacles in the treatment of pancreatic cancer is tumor-associated fibrosis, which limits the penetration and efficacy of therapeutics. αSMA was used as a fibrosis marker associated with senescent cancer-associated fibroblasts in the tumor stroma. Notably, SIWA318H was also found to reduce fibrosis in the tumor microenvironment.

Based on the findings of this study, SIWA318H may represent a promising therapeutic candidate for patients with pancreatic cancer, acting both directly—through targeted cytotoxicity—and indirectly, by potentially enhancing the efficacy of other treatments via reduction of pancreatic tumor fibrosis [51]. 

**Table 2:** Anti-AGEs Antibodies and Their Proposed Clinical Significance

Antibody	Potential role
Anti-methyloglyoxal	Potential biomarker for early cancer detection; may also serve as a biomarker of vascular protection
Anti-imidazolone	Measurement of erythrocyte imidazolone levels using ELISA with AG-1; elevated levels observed in patients with diabetes compared to non-diabetic controls
Anti-pentosidine	Reliable measurement of plasma and urinary pentosidine levels; associated with arterial stiffness in hemodialysis patients; urinary levels correlate with grip strength and gait speed
Anti-CML (2D6G)	Sensitive and specific reagent for the clinical assessment of AGE-associated toxicity
Anti-CEL	Immune responses against CEL may contribute to local inflammation and tissue damage in hidradenitis suppurativa

### Methodological Considerations and Limitations

While the reviewed studies highlight the potential of anti-AGEs antibodies, a critical appraisal reveals several recurring methodological limitations that must be addressed to advance the field.

1. Antibody specificity and cross-reactivity.

The structural heterogeneity of AGEs and the potential for cross-reactivity poses a fundamental challenge. As demonstrated by the evolution from 6D12 anti-CML antibody, which cross-reacts with CEL, to the more specific CMS-10 and 2D6G2 clones, the interpretation of early immunohistochemical and ELISA data requires caution [80, 83]. Studies using polyclonal antibodies or incompletely characterized monoclonal antibodies may overestimate the presence of a specific AGEs. Future research must prioritize rigorous antibody validation.

2. Distinguishing pathogenic from protective antibody responses.

An underexplored finding is the divergent clinical associations of IgM and IgG isotypes. For example, higher levels of anti-MGO-B100 IgM were associated with less severe coronary disease, whereas no such protective association was found for IgG [57]. Similarly, elevated anti-CEL-ApoA1 IgM was associated with better cognitive scores in early Alzheimer’s disease [53]. These findings raise an unanswered question whether IgM antibodies reflect a protective clearance mechanism, while class-switched IgG responses reflect a maladaptive or pathogenic process. Most reviewed studies measure total Ig or a single isotype, limiting mechanistic interpretation. Further studies should differentiate between Ig isotypes and subclasses to clarify their distinct roles.

3. Study design and sample size.

The evidence for many antibodies comes from single-center studies with relatively small sample sizes. While these are valuable proof-of-concept investigations, their generalizability is unknown. The lack of independent, external validation cohorts for most biomarkers represents a significant gap between experimental promise and clinical utility.

4. Clinical diagnostic performance - sensitivity, specificity and unmet requirements.

A critical barrier of the clinical translation of anti-AGEs antibodies as biomarkers is the shortage of data on their diagnostic accuracy. While numerous studies report statistically significant differences in mean antibody titers between patient groups and healthy controls, few have evaluated performance metrics essential for clinical decision-making, including sensitivity, specificity, positive predictive value (PPV) and negative predictive value (NPV). A statistically significant difference does not necessarily equate to a clinically useful discriminatory test. A biomarker with highly overlapping distributions between cases and controls may have unacceptable false-positive or false-negative rates despite a low p-value.

Several biological factors inherent to AGEs biology further complicate the establishment of sensitive and specific cut-off values. First, AGEs accumulation and the resulting antibody response are influenced by confounding variables, such as age, glycemic status, renal function or dietary intake, that are rarely accounted for in study designs. A diagnostic threshold established in a cohort of well-controlled diabetic patients without renal impairment may be invalid in an older population with chronic kidney disease. Second, AGEs are implicated in the pathophysiology of multiple chronic diseases simultaneously (e.g. diabetes, atherosclerosis, neurodegeneration), which inherently limits disease specificity. This suggests that these antibodies may be more suitable for prognostic stratification within a known disease population or for monitoring therapeutic response, rather than for de novo diagnosis among unselected patients.

Future research must move beyond association studies and adopt rigorous biomarker development pipelines, including blinded validation in independent, clinically representative cohorts and head-to-head comparisons against established diagnostic standards.

### Summary

Anti-AGEs antibodies represent a heterogeneous group of molecules, which serve as promising tools in both diagnostics and therapy. Engineered monoclonal antibodies provide specific tools for detecting individual AGEs both in tissue and serum, with recent clones overcoming earlier cross-reactivity problems. Their dynamic development highlights their potential as biomarkers for a vast spectrum of diseases. Noteworthy is their potential use as therapeutic agents. Preclinically, the therapeutic antibody SIWA318H has demonstrated remarkable efficacy in pancreatic cancer models. However, the field is constrained by small, cross-sectional studies that lack validation cohorts and established diagnostic performance metrics. Future research must prioritize rigorous, prospective biomarker validation pipelines and controlled therapeutic comparisons against current standard-of-care treatments.

## Data Availability

No datasets were generated or analysed during the current study.
